# Generalizable MRI normative modelling to detect age-inappropriate neurodegeneration

**DOI:** 10.1186/s13195-025-01872-x

**Published:** 2025-11-12

**Authors:** Thomas D. Parker, Richard A. I. Bethlehem, Jakob Seidlitz, Simon R. White, Michael C. B. David, Magdalena A. Kolanko, Joshua D. Bernstock, Lena Dorfschmidt, Niall Bourke, Anastasia Gailly de Taurines, Jessica A. Hain, Martina Del Giovane, Neil S. N. Graham, Karl A. Zimmerman, Ethan J. F. Losty, Michael Schöll, Meera Srikrishna, Paresh A. Malhotra, Maneesh C. Patel, Gregory Scott, Aaron F. Alexander-Bloch, Edward T. Bullmore, David J. Sharp, the Lifespan Brain Chart Consortium

**Affiliations:** 1 Department of Brain Sciences. Burlington Danes, Imperial College London, The Hammersmith Hospital, London, W12 0HS UK; 2 UK Dementia Research Institute Centre for Care Research and Technology, 9th Floor Sir Michael Uren Hub, Imperial College London White City Campus, 86 Wood Ln, London, W12 0BZ UK; 3 Dementia Research Centre, UCL Queen Square Institute of Neurology, 8-11 Queen Square, London, WC1N 3AR UK; 4 Department of Psychology, University of Cambridge, Downing Pl, Cambridge, CB2 3EB UK; 5 The Lifespan Brain Institute of The Children’s Hospital of Philadelphia and Penn Medicine, Philadelphia, PA 19104 USA; 6 Department of Psychiatry, University of Pennsylvania, Philadelphia, PA 19104 USA; 7 Department of Child and Adolescent Psychiatry and Behavioral Science, The Children’s Hospital of Philadelphia, Philadelphia, PA 19104 USA; 8 MRC Biostatistics Unit, University of Cambridge, Cambridge, CB2 0SR UK; 9 Department of Neurosurgery, Brigham and Women’s Hospital, Harvard Medical School, Boston, MA 02115 USA; 10 David H. Koch Institute for Integrative Cancer Research, Massachusetts Institute of Technology, Cambridge, MA 02139 USA; 11 Department of Neuroimaging, King’s College London, De Crespigny Park, London, SE5 8AF UK; 12 Wallenberg Centre for Molecular and Translational Medicine, University of Gothenburg, 405 30 Gothenburg, Sweden; 13 Department of Psychiatry and Neurochemistry, Institute of Physiology and Neuroscience, University of Gothenburg, 413 45 Gothenburg, Sweden; 14 Department of Neuropsychiatry, Sahlgrenska University Hospital, 413 45 Gothenburg, Sweden; 15 Imperial College Healthcare NHS Trust, Charing Cross Hospital, Fulham Palace Rd, London, W6 8RF UK; 16 Department of Psychiatry, University of Cambridge, Cambridge, CB2 0SZ UK

**Keywords:** MRI, Alzheimer’s disease, Neurodegeneration, Normative modelling, BrainChart, Frontotemporal lobar degeneration, Dementia

## Abstract

**Background:**

Determining whether MRI brain scans demonstrate atrophy that is beyond “normal for age” is challenging. Automated measurements of structural metrics in individual brain regions have shown promise as biomarkers of neurodegeneration, yet widely available reference standards that aid interpretation at the individual level are lacking. Normative modelling, enabling standardized “brain charts”, represents a significant step in addressing this challenge by generating individualized age- and sex- adjusted centile scores derived from large, aggregated datasets for MRI-derived quantitative metrics.

**Methods:**

Using normative data from 56,173 participants across the life course, we have developed regional cortical thickness and amygdala/hippocampal volume brain charts (adjusted for total intracranial volume) that can be applied at the individual level. At the group level, we investigate whether regional centile scores relate to cognitive performance (mini-mental state examination) and discriminate individuals with neuropathological evidence of Alzheimer’s disease (*n* = 351) from propensity-matched controls from the National Alzheimer's Coordinating Center (NACC) dataset. In addition, we explored the relationships between disease stage, cognition, regional tau deposition and regional centile scores in amyloid-β-PET-positive individuals with Alzheimer’s disease dementia (*n* = 39) and mild cognitive impairment (*n* = 71) from the Alzheimer’s Disease Neuroimaging Initiative-3 (ADNI-3). We then extended this approach to phenotypes of frontotemporal lobar degeneration using the Neuroimaging in Frontotemporal Dementia dataset (*n* = 113).

**Results:**

We demonstrate BrainChart’s application to illustrative individual cases. At the group level, we show that in Alzheimer’s disease, regional centile scores from brain charting predicted cognitive performance, temporal lobe tau PET tracer uptake and discriminated disease groups from propensity matched cognitively normal controls in independent cohorts. Distinct patterns of age-inappropriate cortical atrophy were also evident in different clinical phenotypes of frontotemporal lobar degeneration from the Neuroimaging in Frontotemporal Dementia dataset.

**Conclusions:**

Regional centile scores derived from an extensive normative dataset represent a generalizable method for objectively identifying atrophy in neurodegenerative diseases and can be applied to determine neurodegenerative atrophy at the individual level.

**Supplementary Information:**

The online version contains supplementary material available at 10.1186/s13195-025-01872-x.

## Background

Increasing age is a key determinant of cerebral brain structure [1]. Determining whether a structural magnetic resonance imaging (MRI) brain scan demonstrates a pattern of cerebral atrophy beyond the level that is “normal for age” is an important, yet challenging, component of clinical assessment. In particular, it is often a key step in the evaluation of individuals with cognitive impairment to establish if there is evidence of atrophy consistent with a neurodegenerative process, as well as providing an indicator of overall disease severity [2–4]. In most clinical settings, this determination relies on subjective visual assessment, which can be unreliable and is associated with considerable inter-rater variability [5–8]. Furthermore, there is evidence suggesting that visual assessment in the form of visual atrophy rating scales has limitations in terms of their clinical utility [9]. This is particularly challenging cross-sectionally, where previous imaging is unavailable for comparison and a benchmark specific to the individual concerned is lacking.

Objective quantification of brain structure, using automated techniques such as estimates of regional cortical thickness, hippocampus volume, and amygdala volume, has been a key focus of imaging research. These metrics have been proposed as biomarkers of neurodegeneration to aid stratification of individual patients in conditions such as Alzheimer’s disease (AD) [1, 3, 10–17]. However, widely available reference standards for structural MRI metrics that can be generalized to different settings and applied to the individual level are limited. Quantifying the extent to which an individual differs from a reference population represents a key aspect of addressing this issue, and normative modelling of brain structure across the lifespan has been advanced by several large-scale initiatives with successful application to a variety of central nervous system disorders [18–29]. In particular, there has been work using normative modelling that has enabled eloquent MRI staging of neurodegenerative disorders including AD and Frontotemporal Dementia [25–28].

“BrainChart” (https://github.com/brainchart/Lifespan), is an interactive open source tool, which has amassed one of the largest aggregated published datasets of structural MRI brain scans to date [30]. BrainChart estimates age- and sex-adjusted centile scores for structural MRI metrics using a generalized additive models for location, scale and shape (GAMLSS) approach [30, 31]. Crucially, BrainChart encompasses methodology for out-of-sample centile scoring that accounts for study-specific statistical offsets, enabling application to new datasets, and so offering great potential as a generalizable tool for estimating brain structure in a wide range of settings. Initial work utilizing the BrainChart approach largely focussed on global metrics of brain structure such as total grey matter volume. To extend this work, we have applied this approach to individual brain regions and developed an individualized pipeline to calculate centile scores for regional cortical thickness and hippocampal volume using 70,148 scans from 56,173 participants from across the life course (age range 115 days post conception to 100 years old) as a reference population.

Here, we present case studies to illustrate the potential of this approach at the individual level. Furthermore, as a proof of principle analysis, we investigated the utility of regional BrainChart centile scores as a biomarker of neurodegeneration in human disease in multiple independent datasets. This included participants with neuropathological evidence of AD from the National Alzheimer's Coordinating Center (NACC) dataset, as well as a cohort of cognitively impaired participants with amyloid-β (Aβ) PET evidence of AD from the Alzheimer's Disease Neuroimaging Initiative-3 (ADNI-3) dataset, in order to test the following hypotheses: (1) regional BrainChart derived centile scores would correlate with disease severity estimated by mini-mental state examination (MMSE) score; (2) BrainChart derived regional cortical thickness, hippocampal volume and amygdala volume centile scores would reliably differentiate individuals with AD pathology from cognitively normal controls; and (3) in patients with available tau PET imaging (ADNI-3), estimates of regional tau deposition would predict BrainChart derived regional centile scores. In addition, we extended this approach to a cohort of participants with phenotypic variants of frontotemporal lobar degeneration from the Neuroimaging in Frontotemporal Dementia (NIFD) dataset to test the hypothesis that regional BrainChart derived cortical thickness centile scores could demonstrate distinct patterns of cortical neurodegeneration in individuals presenting with neurodegenerative diseases distinct from typical AD.

## Methods

### Individual case studies

To provide illustrative examples of how age- and sex-adjusted regional centile scores calculated using the BrainChart can be applied at the individual level, we present four case studies where we have implemented the BrainChart out-of-sample estimation tool [30]. Participants were recruited to the ‘Minder’ study, an observational study of community-dwelling people living with dementia run by the Care, Research and Technology Centre of the UK Dementia Research Institute (Health Research Authority’s London-Surrey Borders Research Ethics Committee—19/LO/0102) [32]. MRI data were acquired using a Siemens Verio 3 T scanner equipped with a 32-channel head coil. All MRI brain scans from the case reports were reported by consultant radiologists (including experienced neuroradiologists) employed within the National Health Service and obtained by reviewing each participant's medical health records.

Each participant underwent a T1-weighted, three-dimensional magnetization-prepared rapid acquisition gradient-echo (3D-MPRAGE) sequence. Imaging parameters included: repetition time (TR) = 2300 ms, echo time (TE) = 2.98 ms, inversion time = 900 ms, flip angle = 9°, bandwidth = 240 Hz/pixel, acquisition matrix = 256 × 240 × 160, and isotropic voxel size = 1.0 × 1.0 × 1.0 mm3. The relevant code used to generate individualized BrainChart output is freely accessible and can be downloaded via: https://github.com/brainchart/Lifespan_Imperial.

### Cohorts

#### National Alzheimer’s Coordinating Center (NACC) dataset

Structural MRI data from the NACC dataset was processed using FreeSurfer version 6.0 using both T1‐weighted and T2‐weighted/FLAIR images (https://surfer.nmr.mgh.harvard.edu/fswiki/recon-all#UsingT2orFLAIRdatatoimprovepialsurfaces). 1400 cognitively normal participants (median age = 70.5 years and proportion female = 66.4%) and 351 participants with *post-mortem* evidence of AD pathology were included in the analysis (median age = 78.3 years and proportion female = 43.3%). Participants were defined as cognitively normal based on a clinician diagnosis of “Normal cognition and behaviour” (NORMCOG = 1 in NACC data dictionary) and valid clinical visit and MMSE score obtained within 6 months of their MRI scan. *Post-mortem* evidence of AD pathology was defined as evidence of moderate or frequent CERAD neuritic plaque density and evidence of neurofibrillary deposition (Braak stage between III and VI) [33].

#### Alzheimer's Disease Neuroimaging Initiative-3 (ADNI-3) dataset

Data used in the preparation of this article were obtained from the Alzheimer’s Disease Neuroimaging Initiative (ADNI) database (adni.loni.usc.edu). The ADNI was launched in 2003 as a public–private partnership, led by Principal Investigator Michael W. Weiner, MD. The primary goal of ADNI has been to test whether serial MRI, PET, other biological markers, and clinical and neuropsychological assessment can be combined to measure the progression of MCI and early AD. For up-to-date information, see www.adni-info.org. Cross-sectional structural MRI data processed by University of California, San Francisco using FreeSurfer version 6.0 and its associated data dictionary were downloaded from the ADNI database (“UCSF—Cross-Sectional FreeSurfer (6.0), Version: 2022–08–17”). Only data from scans that were deemed to have passed visual quality control by UCSF and had valid diagnosis and MMSE score data obtained within 6 months of their MRI scan were used. Aβ and tau PET data processed by University of California, Berkeley was also downloaded from the ADNI database (“UC Berkeley—Amyloid PET 6 mm Res analysis, Version: 2023–12-04”; and “UC Berkeley—Tau PET PVC 6 mm Res analysis, Version: 2023–12-04”). The Aβ PET tracers used were [^18^F]florbetaben and [^18^F]florbetapir. Standardized uptake value ratio (SUVR) for each tracer were calculated across a cortical summary region using a combination of frontal, temporal, parietal, cingulate regions from the Desikan-Killiany cortical atlas with the whole cerebellum as a reference. Participants were binarized into positive or negative groups based on a tracer dependent SUVR cut-off (1.08 for florbetaben and 1.11 for florbetapir). All SUVRs were converted to Centiloid values to enable comparability between Aβ PET tracer data. Tau PET data were obtained using [^18^F]flortaucipir. Regional SUVR values, with partial volume correction, for individual regions from the Desikan-Killiany cortical atlas were derived following intensity normalisation using inferior cerebellar grey matter as a reference region. Hippocampal tau PET data were not used due to concerns of contamination by off-target binding of flortaucipir in the choroid plexus. There were 39 Aβ-positive participants with a diagnosis of AD dementia (median age = 75.0 years and proportion female = 35.9%), 71 Aβ-positive participants with a diagnosis of mild cognitive impairment (MCI) (median age = 74.7 years and proportion female = 53.5%) and 247 Aβ-negative cognitively normal participants (median age = 70.8 years and proportion female = 57.9%) available for analysis.

#### Frontotemporal lobar degeneration neuroimaging initiative (NIFD) dataset

The Frontotemporal lobar degeneration neuroimaging initiative (NIFD) was funded through the National Institute of Aging, and started in 2010 to identify neuroimaging modalities and methods of analysis for tracking FTLD and to assess the value of imaging versus other biomarkers in diagnostic roles. The Principal Investigator of NIFD was Dr. Howard Rosen, MD at the University of California, San Francisco. The data are the result of collaborative efforts at three sites in North America. For up-to-date information on participation and protocol, please visit http://memory.ucsf.edu/research/studies/nifd. Cross-sectional structural T1-weighted MRI data was downloaded from the NIFD website and processed using FreeSurfer version 6.0.0. All scans and segmentations included passed quality control following visual inspection. Data were available for 113 cognitively normal participants (median age = 64 years and proportion female = 59.3%); 49 participants with a clinical diagnosis of behavioural variant frontotemporal dementia (bvFTD) (median age = 61 years and proportion female = 36.7%); 32 participants with a clinical diagnosis of semantic variant primary progressive aphasia (PPA-SV) (median age = 63.5 years and proportion female = 43.8%); and 32 participants with a clinical diagnosis of progressive nonfluent variant primary progressive aphasia (PPA-PNFA) (median age = 68 years and proportion female = 56.3%).

### Centile score calculation

Values for regional cortical thickness and hippocampal/amygdala volume measurements were extracted from the “aparc.stats” and “aseg.stats” files for each individual following Freesurfer processing. Calculating centile scores requires estimating a normative reference model. Following Bethlehem, Seidlitz, White et al., (2022), a normative GAMLSS model was estimated from population reference data consisting of 56,173 scans from health controls across the lifespan [30]. Estimated total intracranial volume (eTIV) is an important confound in volumetric studies of brain structure [30, 34]. To address this, new models for hippocampal and amygdala volume were generated to account for the non-linear relationships between brain volume and eTIV across the life course, as well as adjusting for age and sex. These non-linear age effects were modelled using fractional polynomial transformations within the GAMLSS framework, which allows flexible modelling of location, scale, and shape parameters. Hippocampal and amygdala models included an interaction term between scanning site and FreeSurfer software version, rather than treating these variables independently, to better account for combined site-specific processing effects on regional volumetric measures. Model building followed previously published procedures, with iterative evaluation of model fit and residual diagnostics to ensure robust normative trajectories adjusted for technical variability and biological confounds [30]. Each brain region was modelled independently, with region-specific smoothing parameters optimized separately using generalized cross-validation.

None of the data used in this study (NACC, ADNI, NIFD) were used to estimate the reference model. Thus, to obtain centiles for these new studies required performing out-of-sample estimation of study-specific random effects (following the previously published methods using maximum likelihood estimation), which relies on imaging data from cognitively normal participants (https://github.com/brainchart/Lifespan?tab=readme-ov-file#out-of-sample-estimation-−350-novel-script---si-18-) [30].

**Fig. 1:**
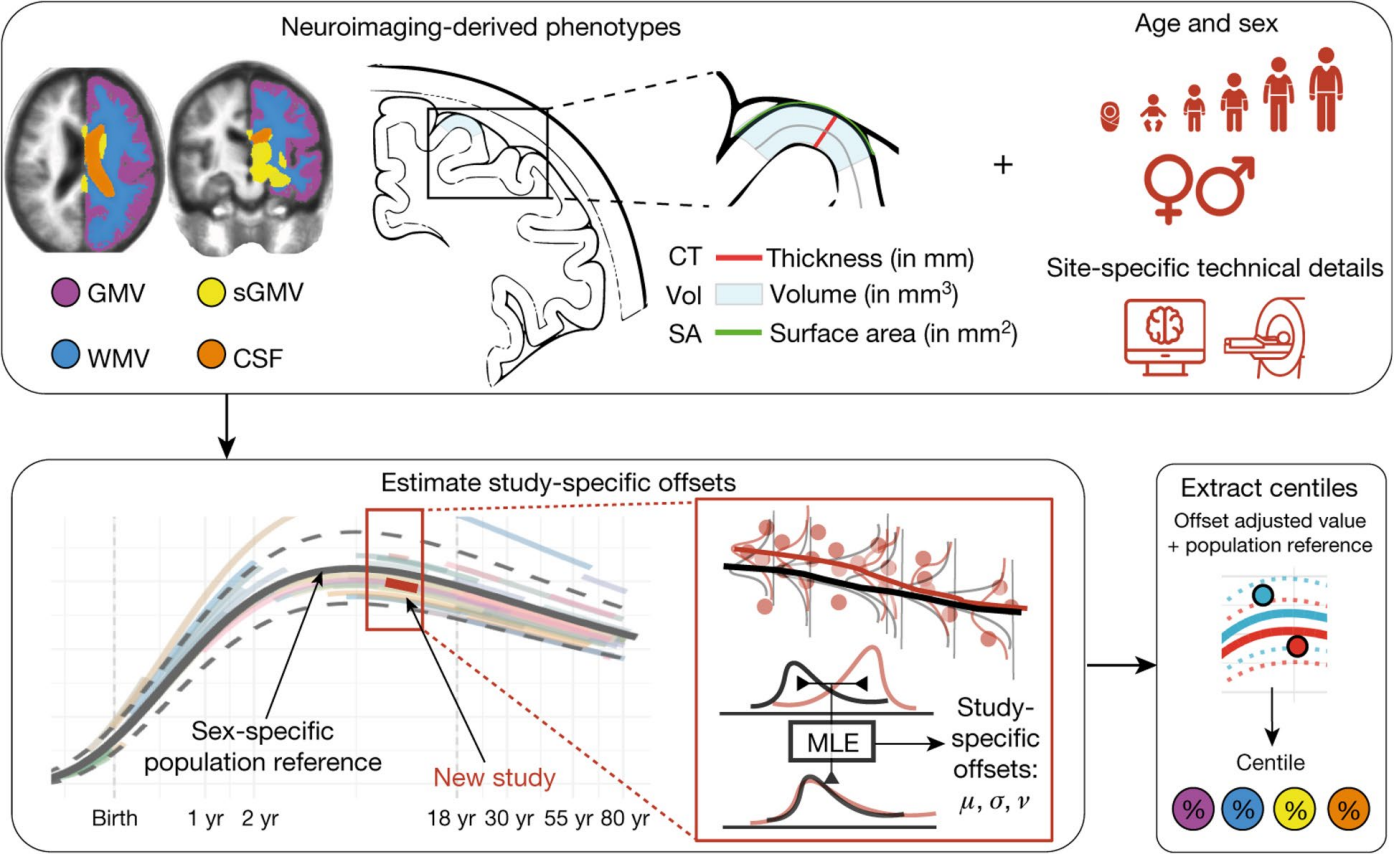
Brain phenotypes were measured in a reference dataset of MRI scans. Generalized Additive Models for Location, Scale, and Shape (GAMLSS) were used to estimate the relationship between MRI structural metrics and age, stratified by sex, and adjusted for technical and other sources of variation across scanning sites and studies. The normative trajectory, showing the median and confidence intervals for each phenotype is plotted as a population reference curve. Out-of-sample data from a new MRI study are aligned to the corresponding age range of this trajectory using maximum likelihood estimation to estimate study-specific offsets (random effects) for the mean (μ), variance (σ), and skewness (ν) of the statistical distribution, considering age and sex. This alignment allows centile scores to be calculated for each scan on the same scale as the reference population, accounting for study-specific batch effects. Reproduced from Bethlehem, Seidlitz, White et al., Brain charts for the human lifespan [30]. Reproduced under a Creative Commons Attribution 4.0 International License (http://creativecommons.org/licenses/by/4.0/)

This method aligns new study data with the established normative trajectory by estimating study-specific random effects through maximum likelihood estimation, based on imaging data from cognitively normal participants. This alignment accounts for systematic differences in mean, variance, and skewness that may arise due to demographic or scanner-related factors, enabling centile scores that are comparable to the reference population. Validation studies have demonstrated that stable and unbiased centile estimation requires approximately 100 cognitively healthy participants scanned on the same scanner [30]. A schematic from the original BrainChart paper highlighting the overview of the out-of-sample approach is displayed in Fig. 1.

Using the out-of-sample study-specific random effects and normative GAMLSS model, we can calculate age-, sex-, and eTIV-adjusted centiles for scans in this study (i.e. from NACC, ADNI, and NIFD). For individuals from the ‘Minder’ study, scanner specific random-effects were calculated using the BrainChart out-of-sample estimation tool using 212 scans from cognitively normal participants (median age = 31 years and proportion female = 36.8%) scanned on the same scanner.

Centile scores were calculated for: cortical thickness estimates of individual cortical regions from the left and right cerebral hemispheres from the Desikan-Killiany cortical atlas (“aparc” files); and hippocampal volume estimates for the left and right hemisphere using the subcortical segmentation atlas included in the FreeSurfer recon-all workflow (“aseg” files).

### Statistical analysis

To assess the relationship between regional centile scores and MMSE score, Spearman rank correlations were performed for all 68 regions from the Desikan-Killiany cortical atlas, left and right hippocampi and left and right amygdalae. Analyses were corrected for multiple comparisons using Benjamini–Hochberg methodology across the 72 regions. These analyses were restricted to participants with pathological evidence of Alzheimer’s disease from the NACC dataset and Aβ-positive MCI or AD dementia participants within the ADNI-3 dataset.

For AD related analyses (NACC/ADNI-3), area under the receiver operating characteristic curve (AUC) analyses were performed for a priori regions of interest (ROI) known to be key sites of atrophy in AD, to assess discrimination of patient groups from propensity-matched cognitively normal participants. The ROIs selected were amygdala and hippocampal volume, as well as entorhinal, inferior temporal, middle temporal, precuneus and inferior parietal cortices. This selection was based on previous large scale analyses that evaluated regional cortical thickness and volume methods for measuring AD severity [4]. Logistic regression models were generated for each a priori region individually, as well as all a priori regions combined into a single model. A sensitivity analysis for the NACC dataset using data from participants with Euler index values less than 2 median absolute deviations above the study median was performed to investigate the influence of scan and segmentation quality on the results. The Euler index quantifies topological defects and holes in the surface reconstruction of both hemispheres as a proxy for segmentation quality [35]. To assess the generalizability and robustness of our classification models, we implemented tenfold repeated cross-validation using the caret package in R.

Given the strong relationship between confounding factors such as age and sex on brain structure, in order to investigate the benefit of converting raw regional cortical thickness, amygdala and hippocampal volume estimates into centile scores, similar AUC analyses were performed using raw uncorrected estimates from FreeSurfer output to compare performance with BrainChart centile scores.

Sensitivity and specificity for discriminating patient groups from cognitively normal participants at different cut-points were calculated using the cutpointr package in R. Cut-points that provided the optimal balance between specificity and sensitivity were determined using Youden’s index. Due to significant differences in sex distribution (determined by Chi-squared testing) and age at time of scanning (determined by the Mann–Whitney U test) between patient groups from cognitively normal participants, a nearest neighbour propensity score matching approach based on age and sex was used to generate an age and sex matched control groups using the MatchIt package in R.

To assess the relationship between regional centile scores and regional tau deposition, Spearman rank correlations between regional SUVR values and the respective regional cortical thickness centile score for all 68 regions from Desikan-Killiany cortical atlas and left and right amygdalae were estimated. Similarly to the MMSE score analyses, correction for multiple comparisons using Benjamini–Hochberg methodology across the 70 regions was performed. These analyses were restricted to Aβ-positive MCI or AD dementia participants with available flortaucipir data within the ADNI-3 dataset.

To test the hypothesis that phenotypic variants of frontotemporal lobar degeneration (NIFD) may show distinct patterns of cortical atrophy from each other, AUC analyses were performed assessing the ability of each cortical region centile score to discriminate each phenotypic sub-group (bvFTD, PPA-SV, PPA-PNFA) from propensity-matched controls for all 72 regions of interest.

Median centile scores for each region in each participant group of interest, as well as Spearman’s Rho and associated adjusted p-values for each region in the MMSE score and tau PET analyses, were visualized using the ggseg R package (https://github.com/ggseg/ggseg) [36].

## Results

### Regional BrainChart centile scores can be applied at the individual level

**Fig. 2:**
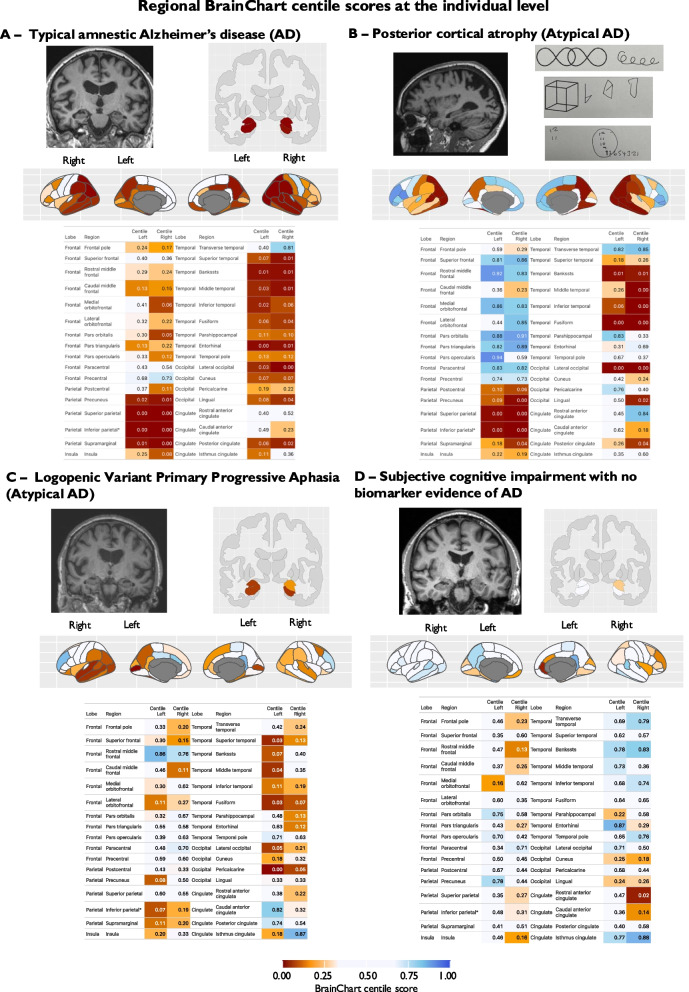
Ilustrative examples of regional BrainChart centile scores applied to the individual level. Regional cortical thickness centile scores are displayed on an inflated brain and in tabular format (cortical regions arranged by cortical lobe - https://surfer.nmr.mgh.harvard.edu/fswiki/CorticalParcellation). **A** A 62-year-old male with young onset amnestic Alzheimer’s disease. A coronal slice from a T1 weighted MRI scan and hippocampal volume centile scores are shown. **B** A 59-year-old female with posterior cortical atrophy. Top row shows sagittal slice of T1 weighted MRI scan and visuospatial tasks from the Addenbrooke's Cognitive Examination-III. **C** 74-year-old female diagnosed with logopenic variant of primary progressive aphasia. **D** A 60-year-old male with subjective cognitive complaints, normal cognitive testing performance and no evidence of Alzheimer’s disease on blood and CSF biomarker testing. Key: AD = Alzheimer’s disease

Figure 2 shows illustrative examples of BrainChart applied to individuals presenting with cognitive complaints. Panel A demonstrates a 62-year-old male with progressive memory difficulties and a clinical diagnosis of Alzheimer’s disease. MMSE score was 20/30. Available cerebrospinal fluid (CSF) biomarkers were supportive of Alzheimer’s pathology (elevated CSF total tau 1021 pg/ml (146–595) and low CSF Abeta 1–42 316 pg/ml (627–1322)). Plasma phosphorylated tau 217 level performed for research purposes was significantly elevated at 1.18 pg/ml [37]. Clinical radiological review stated that the MRI brain scan demonstrated normal appearances for age. However, BrainChart revealed evidence of age-inappropriate atrophy in a range of regions, most prominently in temporal and parietal lobes, but also some frontal regions, with relative sparing of sensorimotor regions. Panel B shows data from a 59-year-old female with progressive memory complaints, but also progressive visuospatial, visuoperceptual deficits, homonymous hemianopia, impaired colour vision and normal visual acuity. The Addenbrooke's Cognitive Examination-III score was 50/100 (attention 11/18; memory 10/26; fluency 6/14; language 8/26; visuospatial 5/16). CSF biomarkers were also supportive of Alzheimer’s pathology (elevated CSF total tau = 1953 pg/ml (146–595) and low Abeta1-42/1–40 ratio = 0.027 (< 0.065)). The MRI brain was originally reported as normal for age. BrainChart again revealed evidence of age-inappropriate atrophy in temporal and parietal lobes (although medial temporal structures such as entorhinal cortex and parahippocampus are relatively spared) with marked sparing of frontal regions, consistent with a diagnosis of posterior cortical atrophy. Panel C reveals data from a 74-year-old female with progressive language difficulties with relatively sparing of episodic memory ultimately diagnosed with logopenic variant of primary progressive aphasia. Plasma Phosphorylated tau 217 levels performed for research purposes were elevated at 1.81 pg/ml [37]. Again, visual read of the MRI brain scan was felt to be within normal limits. BrainChart revealed evidence of age-inappropriate atrophy, particularly in left tempo-parietal regions. Panel D reveals evidence of a 60-year-old male with subjective cognitive complaints, who was provisionally diagnosed with Alzheimer’s disease following a MRI brain scan report stating that imaging revealed greater than expected medial temporal and neocortical volume loss for age prior to specialist assessment. There was subsequently limited progression in symptoms and an Addenbrooke's Cognitive Examination-III score was 98/100. In addition, formal neuropsychological testing did not identify any definite cognitive deficit. CSF biomarkers were not supportive of Alzheimer’s pathology (normal CSF total tau = 144 pg/ml (146–595) and normal Abeta1-42/1–40 ratio = 0.068 (< 0.065)). Plasma phosphorylated tau 217 levels performed for research purposes were normal at 0.20 pg/ml [37]. BrainChart did not reveal a pattern of atrophy consistent with Alzheimer’s disease, in line with the fluid biomarkers and clinical course.

### Regional BrainChart centile scores correlate with cognitive performance and discriminate individuals with pathological Alzheimer’s disease from propensity-matched cognitively normal controls

**Fig. 3:**
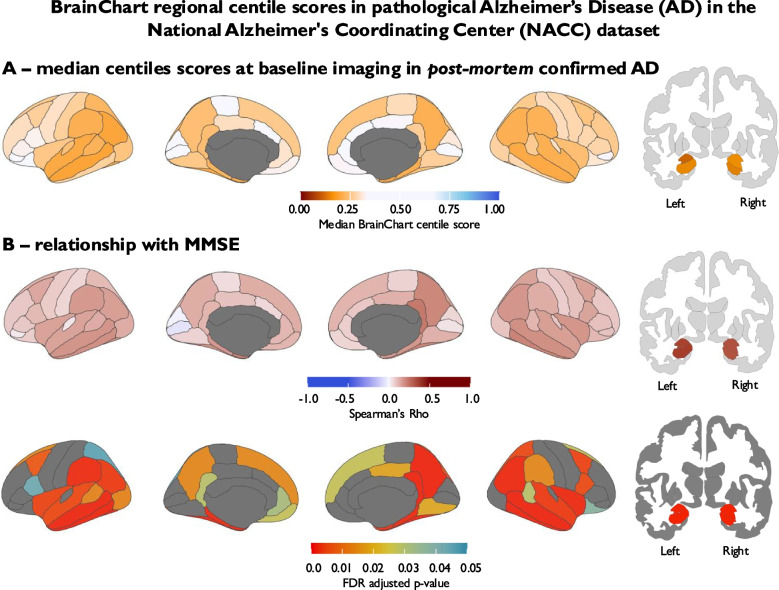
BrainChart median cortical thickness, hippocampal and amygdala volume centile scores derived from baseline MRI brain scans in pathologically confirmed Alzheimer’s disease (**A**) (moderate or frequent CERAD neuritic plaque density + Braak stage III-VI); *n* = 351; Median age = 78.3 years, Median MMSE score = 24/30; Female = 43.3%; Median time to death = 5.9 years. **B** Spearman’s correlation between BrainChart cortical thickness, hippocampal and amygdala volume centile scores and MMSE score. Only *p*-values that were significant following FDR correction are shown. Key: AD = Alzheimer’s disease, FDR = False Discovery Rate, MMSE = Mini-mental state examination, NACC = National Alzheimer's Coordinating Center

**Fig. 4:**
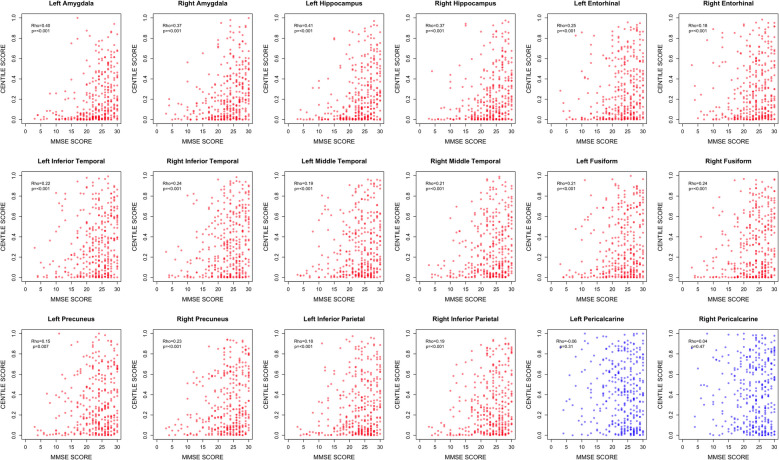
Scatter plots highlighting relationship between BrainChart centile scores and MMSE score in Alzheimer’s disease signature regions (a priori regions of interest—red) and primary visual cortex (control region – blue) in pathologically confirmed Alzheimer’s disease (moderate or frequent CERAD neuritic plaque density + Braak stage III-VI; *n* = 351; Median age = 78.3 years, Median MMSE score = 24/30; Female = 43.3%; Median time to death = 5.9 years). Key: MMSE = Mini-mental state examination

To establish the utility of regional BrainChart scores as a biomarker of neurodegeneration in AD, we investigated first the relationship between regional BrainChart centiles and cognitive performance (MMSE score) in patients with pathologically confirmed AD from the NACC dataset, who were scanned on average approximately 6 years prior to death. In this cohort, the ROIs demonstrating the strongest relationship between regional BrainChart centile score and MMSE score were left hippocampal volume (Spearman’s Rho = 0.41, corrected p-value = 7.64 × 10⁻14), left amygdala volume (Spearman’s Rho = 0.40, corrected p-value = 2.75 × 10⁻13), right amygdala volume (Spearman’s Rho = 0.37, corrected p-value = 2.21 × 10⁻11), and right hippocampal volume (Spearman’s Rho = 0.36, corrected p-value = 3.24 × 10⁻11). A wide range of cortical regions were also positively correlated with MMSE score following correction for multiple comparison testing, largely in frontal, temporal and parietal regions (Spearman’s Rho ranging from 0.11—0.26 in regions with statistically significant correlations), with relative sparing of the rolandic and primary visual areas (Figs. 3 and 4).

**Fig. 5:**
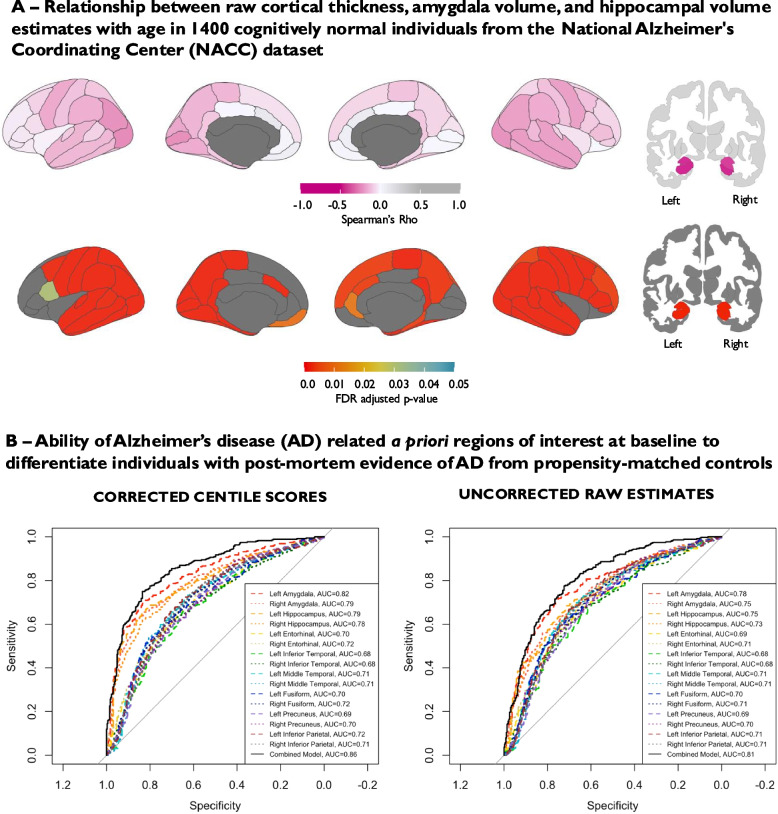
**A** Relationship between age and raw uncorrected estimates of cortical thickness, amygdala volume and hippocampal volume in 1400 cognitively normal individuals (median age = 70.5 years, Median MMSE score = 29/30; Female = 66.4%). **B** Ability of BrainChart centile scores for AD signature regions to discriminate between 351 pathologically confirmed AD cases (moderate or frequent CERAD neuritic plaque density + Braak stage III-VI; Median age = 78.3 years; Median MMSE score = 24/30; Female = 43.3%; Median time to death = 5.9 years) and 351 propensity-matched cognitively normal controls with and without conversion to BrainChart regional centile scores (AUC = area under curve). Centile scores for a priori regions of interest making up the combined model include hippocampal volume, as well as thickness entorhinal, inferior temporal, middle temporal, precuneus and inferior parietal cortices. Key: AD = Alzheimer’s disease, AUC = Area under the curve, FDR = False Discovery Rate, MMSE = Mini-mental state examination, NACC = National Alzheimer's Coordinating Center

In terms of diagnostic ability, amygdala (left AUC = 0.82; right AUC = 0.79) and hippocampal volume (left AUC = 0.79; right AUC = 0.78) centile scores adjusted for age, sex and eTIV were the ROIs that demonstrated the greatest discriminative ability between pathological AD and cognitively normal controls. AD cortical thickness signature region age- and sex-adjusted centile scores AUCs ranged from 0.68—0.72. A combined model containing amygdala, hippocampal volume centile scores and AD cortical thickness signature region centile scores provided an AUC of 0.86 (Fig. 5B). Sensitivity analyses using data from participants with Euler index values less than 2 median absolute deviations above the study median are displayed in supplementary Table 1 and revealed very marginal differences in the results (e.g. combined AUC of 0.87 compared to 0.86 in the complete case analysis) suggesting segmentation quality was unlikely to be driving the results observed. To further validate the robustness of our classification model in the NACC dataset, we implemented tenfold cross-validation using a combined logistic regression model incorporating all AD signature regions. This approach yielded a mean AUC of 0.84, with a sensitivity of 0.76 and specificity of 0.77, indicating strong discriminative performance. These results are consistent with the complete-case AUC estimates and support the reliability of the model in distinguishing individuals with pathological AD from propensity-matched cognitively normal controls.

The importance of correcting for age is highlighted in Fig. 5. As expected, in 1400 cognitively normal participants from the NACC dataset, raw cortical thickness estimates, amygdala and hippocampal volume were strongly related to age at time of scanning (Fig. 5A). When performing AUC analyses using raw cortical thickness estimates and raw amygdala and hippocampal volume, discriminative ability was lower than when using BrainChart centile scores (Fig. 5B). The AUC for a combined model containing raw amygdala volume, raw hippocampal volume and raw cortical thickness estimates of AD-related regions of interest was 0.81, which was lower than the aforementioned AUC of 0.86 obtained when utilizing BrainChart centile scores for the same regions corrected for age and sex (as well as eTIV for amygdala/hippocampal volume), with a statistically significant difference confirmed by DeLong’s test (Z = −4.4, *p* < 0.001; 95% CI for AUC difference: −0.062 to −0.024).

Supplementary Table 2 lists the ranking the AUC of all cortical FreeSurfer regions in terms of their ability to discriminate pathologically confirmed AD from matched controls. Optimal centile cut-points for individual AD signature regions are displayed Supplementary Table 3. The median threshold that best differentiated participants with pathologically confirmed AD from cognitively normal controls was the 36th centile, with values ranging from the 23rd to the 47th centile. Sensitivities for these optimal cut points ranged from 60 to 73%, and specificities ranging from 58 to 83% (Supplementary Table 3). Using progressively lower centile scores increased specificity (scores below the first centile 93% to 98% specific) but decreased sensitivity (scores below the first centile 7% to 20% sensitive) (Supplementary Table 3).

### Regional BrainChart centile scores correlate with disease stage, cognition and regional tau deposition in ADNI-3

**Fig. 6:**
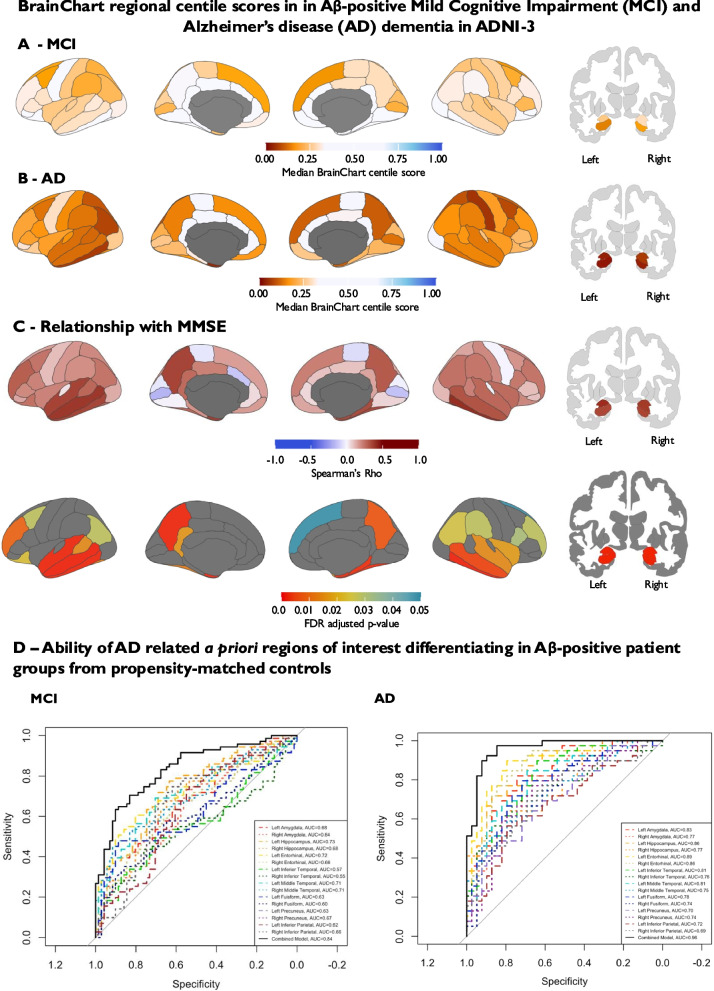
**A** BrainChart median cortical thickness and hippocampal centile scores derived from baseline MRI brain scans in Aβ-positive MCI (*n* = 71; Median age = 74.7 years; Median MMSE = 27/30; Female = 53.5%; Median Centiloid scale score = 82); **B** BrainChart median cortical thickness and hippocampal centile scores derived from baseline MRI brain scans Aβ-positive AD dementia (*n* = 39; Median age = 75.0 years; Median MMSE = 23/30; Female = 35.9%; Median Centiloid scale score = 96). **C** Spearman’s correlation between BrainChart cortical thickness and hippocampal volume centile scores and MMSE score at baseline in Aβ-positive MCI and AD dementia in ADNI-3 (*n* = 110; Median age = 74.8 years; Median MMSE = 27/30; Female = 47.3% Median Centiloid scale score = 87). **D** Ability of BrainChart centile scores for AD signature regions to discriminate between 71 Aβ-positive MCI cases and 71 Aβ-negative propensity-matched cognitively normal participants. **E** Ability of BrainChart centile scores for AD signature regions to discriminate between 39 Aβ-positive AD dementia cases and 39 Aβ-negative propensity-matched cognitively normal participants. Centile scores for a priori regions of interest making up the combined model include hippocampal volume, as well as thickness entorhinal, inferior temporal, middle temporal, precuneus and inferior parietal cortices. Key: Aβ = beta-amyloid, AD = Alzheimer’s disease, ADNI = Alzheimer's Disease Neuroimaging Initiative, FDR = False Discovery Rate, MMSE = Mini-mental state examination

To confirm our findings in a separate independent cohort of individuals with biomarker-defined AD, we also investigated a cohort of participants who had contemporaneous markers of AD pathology in the form of Aβ PET scans from the ADNI-3 cohort. Across all 110 Aβ-positive participants with a diagnosis of MCI or AD dementia in the ADNI-3 cohort a similar pattern of positive correlation between MMSE score regional BrainChart centile scores was evident (Fig. 6C). In the a priori AD-related regions of interest, BrainChart centile score AUCs for discriminating Aβ-positive MCI participants and propensity-matched Aβ-negative cognitively normal controls in ADNI ranged from 0.55—0.73, whilst the combined model of all regions provided an AUC of 0.84. A combined model containing raw amygdala volume, raw hippocampal volume and raw cortical thickness estimates was 0.82, but this difference was not statistically significant (Z = 0.7, *p* = 0.44; 95% CI for AUC difference: −0.023 to 0.054). Tenfold cross-validation using a combined logistic regression model incorporating all AD signature regions yielded a more conservative mean AUC of 0.74, with a sensitivity of 0.66 and specificity of 0.67. The discrepancy between complete-case and cross-validated AUC estimates may be attributable to the relatively small sample size, which can lead to overfitting in the full model and increased variability in resampling-based validation procedures.

Regional BrainChart centile scores demonstrated much greater ability to discriminate Aβ-positive AD dementia participants from Aβ-cognitively normal controls with AUCs for the individual regions ranging from 0.69 to 0.89 and a combined model of all regions providing an AUC of 0.96 (Fig. 6D). A combined model containing raw amygdala volume, raw hippocampal volume and raw cortical thickness estimates was 0.97 and was not statistically significantly different from the BrainChart model (Z = −0.96, *p* = 0.33; 95% CI for AUC difference: −0.042 to 0.014). Tenfold repeated cross-validation using a combined logistic regression model incorporating all AD signature regions again yielded a more conservative mean AUC of 0.85, with a sensitivity of 0.76 and specificity of 0.75 in keeping with performance in the NACC dataset. Again, this discrepancy may be related to the relatively small sample size in the ADNI analysis.

**Fig. 7:**
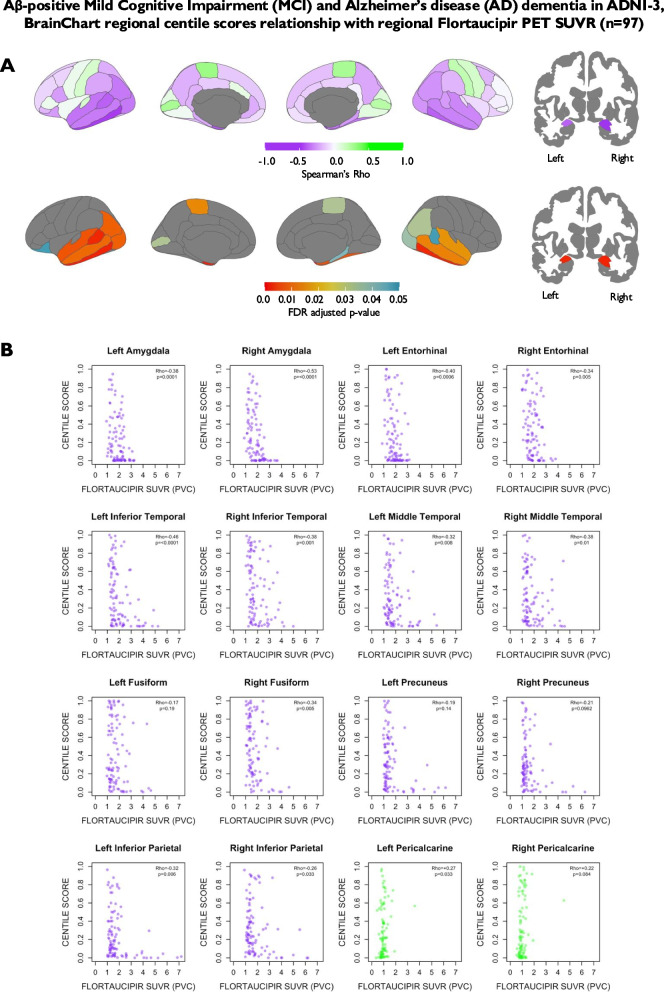
**A** Spearman’s correlation between BrainChart centile scores and regional tau PET (PET ligand = flortaucipir) SUVR (partial-volume-corrected with the inferior cerebellar grey matter as the reference region) at baseline in Aβ-positive MCI and AD dementia cases in ADNI-3 (*n* = 97). NB Hippocampus not examined due to flortaucipir signal in hippocampus being contaminated by off-target binding in the choroid plexus [38]. B. Scatter plots between highlighting relationship between BrainChart centile scores and Tau PET SUVR in Alzheimer’s disease signature regions (purple) and primary visual cortex (control region – green). Key: Aβ = beta-amyloid, AD = Alzheimer’s disease, ADNI = Alzheimer's Disease Neuroimaging Initiative, FDR = False Discovery Rate, MCI = Mild Cognitive Impairment, SUVR = standard uptake value ratio for individual regions of interest derived following intensity normalisation using inferior cerebellar grey matter as a reference region

There were 97 Aβ-positive participants with a diagnosis of MCI or AD dementia in the ADNI-3 cohort with tau PET data. Regional BrainChart centile scores were negatively correlated with regional partial volume corrected flortaucipir SUVR values primarily in the amygdala and temporal cortical regions following correction for multiple comparisons, with Spearman’s Rho values ranging from −0.25 to −0.53 in regions with statistically significant correlations (Fig. 7). There was a positive correlation (i.e. the opposite effect seen in temporal regions) between centile scores and flortaucipir SUVR in the paracentral lobule and right pericalcarine cortex, although the biological relevance of this unclear as these are locations not typically implicated in AD pathophysiology and likely driven by single outlier (Fig. 7).

### Regional BrainChart centile scores demonstrate distinct patterns of cortical atrophy in phenotypic variants of frontotemporal lobar degeneration

**Fig. 8:**
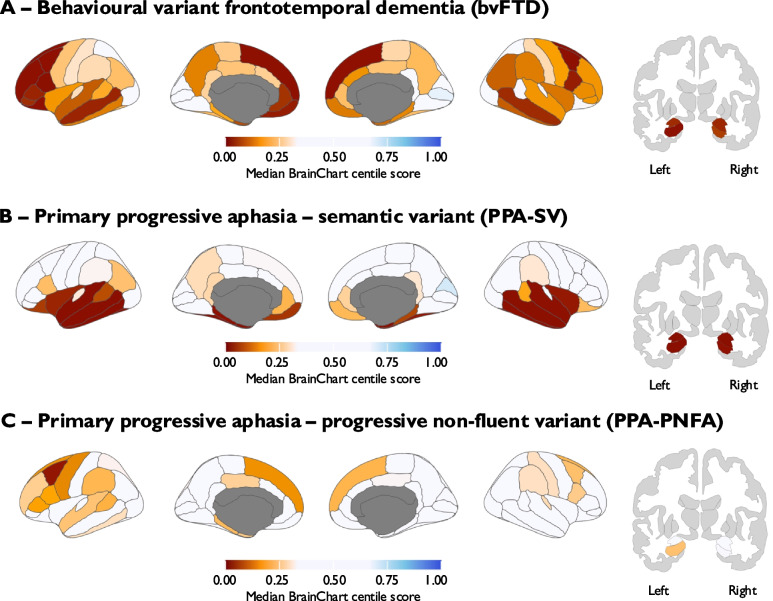
**A** BrainChart median cortical centile scores derived from baseline MRI brain scans in patients with behavioural variant frontotemporal dementia (*n* = 49; Median age = 61 years; Female = 36.7%); **B** BrainChart median cortical centile scores derived from baseline MRI brain scans in patients with primary progressive aphasia semantic variant (*n* = 32; Median age = 63.5 years; Female = 43.8%); **C** BrainChart median cortical centile scores derived from baseline MRI brain scans in patients with primary progressive aphasia non-fluent variant (*n* = 32; Median age = 68 years; Female = 56.3%)

In order to demonstrate that BrainChart regional centile scores could identify distinct patterns of cortical neurodegeneration in individuals presenting with neurodegenerative diseases distinct from typical AD, we extended this approach to a cohort of participants with phenotypic variants of frontotemporal lobar degeneration from NIFD, which includes 49 participants with a clinical diagnosis of bvFTD, 32 participants with a diagnosis of PPA-SV and 32 participants with a clinical diagnosis of PPA-PNFA. Median BrainChart cortical thickness centile scores for three phenotypes are displayed visually in Fig. 8 and show striking patterns of age inappropriate atrophy consistent with existing literature in these conditions [39, 40]. The top 10 ranking individual regions for discriminating bvFTD from propensity-matched cognitively normal participants using BrainChart cortical thickness centile scores are shown in supplementary Table 4, where AUCs ranged from 0.84 to 0.91. This included several regions that also strongly discriminated AD patients from controls (e.g. hippocampus and middle temporal). To identify which regions may be more specific to bvFTD, we leveraged the common framework that the BrainChart approach provides and investigated which regions best discriminated bvFTD participants from propensity-matched participants from the NACC dataset who ultimately had pathological evidence of AD at post-mortem (supplementary Table 4). The best performing regions (with AUC > 0.7) were all frontal (superior frontal, caudal middle frontal pars opercularis, lateral orbitofrontal, rostral middle frontal, pars orbitalis, pars triangularis and medial orbiotofrontal).

Median BrainChart cortical thickness centile scores for the semantic and progressive non-fluent variants of PPA are displayed in Fig. 8B and C. The top 10 ranking individual regions for discriminating each PPA phenotype from propensity-matched cognitively normal participants is displayed in supplementary Table 5, and the top 10 ranking individual regions for discriminating each PPA phenotype from each other are displayed in supplementary Table 6. AUCs for PPA-SV for discriminating patient from cognitively normal participants and PPA-PNFA in a range of temporal lobe regions such as temporal pole, amygdala, entorhinal and hippocampus were very high, whilst the discriminative ability in the context of PPA-PNFA were more modest, with left frontal regions (pars triangularis, precentral and caudal middle frontal) being most discriminative.

## Discussion

Leveraging the largest known normative dataset of aggregated structural MRI scans currently available, we demonstrate how normative modelling, or “brain charts”, that generate individualized age- and sex-adjusted centile scores for MRI-derived quantitative metrics is an exciting approach that can be applied to individuals presenting with cognitive symptoms. Specifically, in selected individual cases we highlight the translational benefit of this approach by showing how quantitative information regarding brain atrophy can be used to enhance clinical assessment when compared with standard reporting. Furthermore, we show in two distinct cohorts that BrainChart-derived regional cortical thickness, hippocampal and amygdala volume centile scores provide biologically relevant information regarding the extent of neurodegeneration in AD. The feasibility of large-scale lifespan approaches, such as those developed by the ENIGMA Lifespan Working Group has been demonstrated previously, and our work further supports the feasibility of deriving population-representative reference charts in clinical populations [22–24]. In addition, prior work has shown that applying lifespan modelling to AD and FTLD using similar datasets (NACC, ADNI, NIFD) have demonstrated distinct anatomical staging patterns across sporadic and genetic variants of frontotemporal dementia [25–27]. While these studies used similar datasets and modelling principles, our approach extends this with a larger normative dataset and by integrating PET biomarkers of AD pathology as, well as post-mortem confirmed cases and illustrates how such approaches can be applied at the individual level.

Neurodegeneration is felt to be a relatively late event in the pathophysiological continuum of AD and closely related to cognitive impairment [12]. In two independent datasets we show clear evidence that regional BrainChart centile scores correlated with cognition (MMSE score), highlighting the potential of this tool to provide clinically relevant information regarding disease severity. Deposition of neurofibrillary containing tangles, particularly in temporal regions, has shown to be a strong predictor of atrophy in AD [12, 15, 41, 42]. Using ADNI-3 data, our analyses revealed strong associations between BrainChart regional centile scores and tau PET tracer uptake in the temporal lobes, highlighting that regional centile scores provide pathologically relevant information in AD. Furthermore, the ability of regional BrainChart centile scores to discriminate individuals with Aβ-positive AD dementia from controls was greater than the ability of regional BrainChart centile scores to discriminate individuals with Aβ-positive MCI from controls, again highlighting the utility of this approach in assessing disease severity.

In addition, we have provided evidence that regional BrainChart centile scores have utility in identifying age-inappropriate atrophy patterns in other neurodegenerative contexts, namely phenotypic variants of FTLD. In particular, we demonstrate distinct patterns of age-inappropriate atrophy in different phenotypes of primary progressive aphasia, which can be a diagnostically challenging area [39].

Identifying patterns of atrophy that are abnormal for age is a particularly challenging component of subjective radiological assessment and is associated with significant inter-rater reliability [5–8]. Quantitative analysis of structural MRI has been a major component of neurodegenerative research over the past decades and there is a wealth of data demonstrating its utility in a research setting [3, 10–14]. To-date these approaches are not used routinely in clinical settings. Modelling approaches that leverage large normative datasets represent a pathway to bridging this gap and applying such techniques at the individual level [5, 18–21]. The ability to account for age at the individual level is a crucial aspect of our approach. The relationship between age and brain structure and how this differs in neurodegenerative diseases such as AD is well established [1, 17]. As such, age is a vital consideration when assessing a structural MRI scan and this was reflected in the NACC dataset, where, as expected, there were strong relationships between age and regional structural metrics in cognitively healthy individuals [30]. In addition, we showed that using age-corrected regional BrainChart scores provided greater diagnostic accuracy compared to uncorrected raw cortical thickness and hippocampal volume estimates from baseline imaging in terms of their ability to discriminate individuals who ultimately were shown to have AD pathology at post-mortem from propensity-matched cognitively normal controls.

Using a Youden index approach, we identified optimal centile cut-points for individual AD signature regions. The median threshold that best differentiated participants with pathologically confirmed AD from cognitively normal controls was below the 36th centile, with values ranging from the 23rd to the 47th centile (see Supplementary Table 3). These thresholds may offer a useful starting point for identifying regionally specific atrophy that deviates from age-expected norms. However, it is important to emphasize that these values should not be regarded as definitive clinical cut-offs at this stage. Rather, they may help inform further investigation, particularly when low centile scores are observed across multiple regions in a consistent pattern. Notably, very low centile scores were highly specific but demonstrated limited sensitivity, suggesting that while extremely low scores may be particularly informative for confirming pathology, more moderate thresholds may strike a better balance between sensitivity and specificity for broader clinical application. Future prospective studies will be needed to assess the clinical utility of these centile thresholds in real-world settings, especially in relation to diagnostic decision-making and disease staging. Furthermore, integrating longitudinal data into normative models may enhance centile-based interpretation by capturing within-individual trajectories of brain atrophy over time.

Whilst other tools are available that provide automated readouts of brain morphometry, [5, 19, 43, 44] a significant advantage of BrainChart is that it is unique in terms of the magnitude and range of studies contributing to the normative dataset. A further advantage of the BrainChart tool is the functionality of an out-of-sample estimation tool, which allows it to be applied to unique and previously unseen datasets [45]. Here we have demonstrated the power of this approach in this paper by applying the same common framework to multiple independent datasets. This enabled cross-cohort comparisons, highlighted by analyses identifying which regions best differentiates bvFTD (NIFD dataset) from AD (NACC dataset). Critically for its clinical implementation, BrainChart enables application at the individual level and provides a readily interpretable read-out of regional brain structure, which has a range of potential clinical applications, including aiding diagnosis and disease staging. This may be particularly important in the setting of newly emerging disease modifying therapies for AD targeting cerebral Aβ plaques, which are likely to be more efficacious in the earlier stages of AD pathophysiology and normative modelling tools of structural MRI may be able to help predict who is likely to respond to such therapies and aid recruitment to clinical trials [46, 47]. Our approach complements existing tools such as the CentileBrain project, which provides pre-trained regional normative models via an online platform (https://centilebrain.org/), offering an accessible resource for lifespan brain morphometry. Developing such online resources that integrate larger-scale normative datasets and disease-specific information to improve accessibility of these approaches is an important area for future work.

While the out-of-sample estimation tool addresses a significant barrier to implementation of such automatic techniques, there is a requirement for the availability of 100 scans of healthy controls performed on the same scanner to calculate study-specific offsets and implement BrainChart currently, which may limit its application in centres without the requisite number of healthy controls scans. Furthermore, BrainChart currently requires processing with Freesurfer. Freesurfer has been extensively used in the field, however it does have a number of limitations that are important to note. Software version is known to be an important predictor of volume and thickness estimates and the out-of-sample estimation tool includes Freesurfer version as a covariate to account for these version-specific differences [30]. However, recent studies highlight that segmentation differences between versions can be substantial in certain regions. While version adjustment mitigates some of this variability, residual effects may persist and should be considered when comparing across datasets processed with different FreeSurfer versions [48]. Another limitation is that Freesurfer is computationally intensive, which may limit BrainChart’s application more broadly. Future work should explore integrating a wider range of segmentation tools beyond Freesurfer to assess how different processing methods impact normative modeling with BrainChart. This would help identify complementary measures and improve the robustness and generalizability of AD biomarkers across diverse datasets and imaging protocols. While some segmentation methods may offer enhanced accuracy, their computational demands should also be considered to ensure clinical feasibility. Evaluating less-resource-intensive tools alongside more complex ones will be important for developing scalable and effective neuroimaging biomarkers. Broadening segmentation approaches will ultimately enhance the precision and applicability of normative models in AD research [49]. One further potential limitation of normative modelling is that most imaging study participants largely come from more economically developed countries; are more likely to have a white ethnic background; and have higher than average levels of education, which may limit generalizability to more diverse populations [50, 51]. How other neurodegenerative diseases not considered in this analysis such as Parkinson’s disease related dementias, motor neuron disease, chronic traumatic encephalopathy and limbic-predominant age-related TDP-43 encephalopathy should also be the focus of future work [52–55].

Scanner heterogeneity and variation in acquisition protocols across studies contributing to the normative dataset are potential sources of variability. In the original BrainChart work, models that included an interaction term between study and scanning site performed almost identically to those using only a study-level random effect. This is likely because the study-level random effect already captures most of the variability introduced by differences in scanner hardware and acquisition protocols [30]. Similar findings were observed in more recent updates to the regional models [56]. While this suggests such factors have limited impact on the population reference, residual effects at the regional level cannot be completely excluded and should be considered when interpreting results from aggregated multi-site datasets.

While this study applied BrainChart centile scores to cross-sectional imaging data, we acknowledge that longitudinal assessment of brain structure is critical for understanding disease progression in neurodegenerative conditions. An important direction for future work will be the expansion of the BrainChart normative dataset to include more longitudinal data and the development of models that explicitly quantify within-individual change over time. Such models would enable the generation of centile-based trajectories, facilitating the identification of individuals with accelerated atrophy relative to age-expected norms. There is also evidence to suggest that dynamic changes inferred from cross-sectional data may underestimate the degree of change observed in longitudinal measurements. Expanding the normative dataset to incorporate repeated imaging and modelling change within individuals would therefore enhance sensitivity to disease progression and support broader clinical utility of the BrainChart framework [57].

A further important avenue will be to prospectively test its utility in clinical settings, to assess whether provision of regional BrainChart centile score data can aid interpretation and reporting of clinical MRI brain scans. How this intersects with other biomarkers relevant to neurodegenerative disease including newly emerging blood-based biomarkers will be of great utility [37]. Furthermore, adopting a similar BrainChart approach to other imaging modalities commonly used in clinical practice, such as computed tomography, will further enhance its translational potential.

## Conclusions

Regional BrainChart centile scores derived from an extensive normative dataset, can aid in objectively identifying atrophy patterns, region by region, that are unexpected for age. Importantly, this can be applied at the individual level and is easily interpretable, and hence generalizable to variety of settings providing great translational potential in the clinical evaluation of cognitive impairment and dementia.

## Supplementary Information

Supplementary Material 1.

## Data Availability

The National Alzheimer’s Coordinating Center (NACC) database ([https://naccdata.org](https:/naccdata.org)) isavailable to qualified investigators upon data use agreement approval. The Alzheimer’s Disease Neuroimaging Initiative (ADNI) database ([http://adni.loni.usc.edu](http:/adni.loni.usc.edu)) is accessible to researchers following completion of a data use application. The Neuroimaging Frontotemporal Dementia (NIFD) dataset is available through the Frontotemporal Lobar Degeneration Neuroimaging Initiative at https://ida.loni.usc.edu upon registration and approval. Access to data from the Minder study at the UK Dementia Research Institute Centre for Care Research & Technology may be considered for bona fide researchers upon reasonable request. Any data sharing will be subject to appropriate ethical approvals and data use agreements to ensure the privacy and rights of study participants are fully protected. All BrainChart models and code for individualized analysis pipelines can be accessed via: https://github.com/brainchart/Lifespan_Imperial.

## Abbreviations

Aβ: Amyloid-β
AD: Alzheimer’s disease
ADNI-3: Alzheimer’s Disease Neuroimaging Initiative
AUC: Area under the curve
bvFTD: Behavioural variant frontotemporal dementia
CSF: Cerebrospinal fluid
eTIV: Estimated Total Intracranial Volume
FTLD: Frontotemporal lobar degeneration
GAMLSS: Generalized additive models for location, scale and shape
MMSE: Mini-mental state examination
MPRAGE: Magnetization-prepared rapid acquisition with gradient echo
MRI: Magenetic Resonance Imaging
NACC: National Alzheimer's Coordinating Center
NIFD: Neuroimaging in Frontotemporal Dementia
PPA-SV: Semantic variant primary progressive aphasia
PPA-PNFA: Progressive nonfluent variant primary progressive aphasia
ROI: Region of Interest

## References

[1] Fox NC, Schott JM. Imaging cerebral atrophy: normal ageing to Alzheimer’s disease. Lancet. 2004;363(9406):392–4. 15074306 10.1016/S0140-6736(04)15441-X

[2] Harper L, Barkhof F, Scheltens P, Schott JM, Fox NC. An algorithmic approach to structural imaging in dementia. J Neurol Neurosurg Psychiatry. 2014;85(6):692–8. 24133287 10.1136/jnnp-2013-306285 PMC4033032

[3] Jack CR, Wiste HJ, Weigand SD, Knopman DS, Mielke MM, Vemuri P, et al. Different definitions of neurodegeneration produce similar amyloid/neurodegeneration biomarker group findings. Brain. 2015;138(Pt 12):3747–59. 26428666 10.1093/brain/awv283 PMC4655341

[4] Schwarz CG, Gunter JL, Wiste HJ, Przybelski SA, Weigand SD, Ward CP, et al. A large-scale comparison of cortical thickness and volume methods for measuring Alzheimer’s disease severity. NeuroImage: Clinical. 2016;11:802–12. 28050342 10.1016/j.nicl.2016.05.017 PMC5187496

[5] Pemberton HG, Goodkin O, Prados F, Das RK, Vos SB, Moggridge J, et al. Automated quantitative MRI volumetry reports support diagnostic interpretation in dementia: a multi-rater, clinical accuracy study. Eur Radiol. 2021;31(7):5312–23. 33452627 10.1007/s00330-020-07455-8 PMC8213665

[6] Ten Kate M, Barkhof F, Boccardi M, Visser PJ, Jack CR, Lovblad KO, et al. Clinical validity of medial temporal atrophy as a biomarker for Alzheimer’s disease in the context of a structured 5-phase development framework. Neurobiol Aging. 2017;52:167-182.e1. 28317647 10.1016/j.neurobiolaging.2016.05.024

[7] Rhodius-Meester HFM, Benedictus MR, Wattjes MP, Barkhof F, Scheltens P, Muller M, et al. MRI visual ratings of brain atrophy and white matter hyperintensities across the spectrum of cognitive decline are differently affected by age and diagnosis. Front Aging Neurosci. 2017;9(9):117. 28536518 10.3389/fnagi.2017.00117 PMC5422528

[8] Velickaite V, Ferreira D, Lind L, Ahlström H, Kilander L, Westman E, et al. Visual rating versus volumetry of regional brain atrophy and longitudinal changes over a 5-year period in an elderly population. Brain Behav. 2020;10(7):e01662. 32436327 10.1002/brb3.1662 PMC7375085

[9] Loreto F, Gontsarova A, Scott G, Patel N, Win Z, Carswell C, et al. Visual atrophy rating scales and amyloid PET status in an Alzheimer’s disease clinical cohort. Ann Clin Transl Neurol. 2023;10(4):619–31. 36872523 10.1002/acn3.51749 PMC10109315

[10] Dickerson BC, Wolk DA. MRI cortical thickness biomarker predicts AD-like CSF and cognitive decline in normal adults. Neurology. 2012;78(2):84–90. 22189451 10.1212/WNL.0b013e31823efc6c PMC3466670

[11] Dickerson BC, Bakkour A, Salat DH, Feczko E, Pacheco J, Greve DN, et al. The cortical signature of Alzheimer’s disease: regionally specific cortical thinning relates to symptom severity in very mild to mild AD dementia and is detectable in asymptomatic amyloid-positive individuals. Cereb Cortex. 2009;19(3):497–510. 18632739 10.1093/cercor/bhn113 PMC2638813

[12] Jack CR, Wiste HJ, Weigand SD, Therneau TM, Knopman DS, Lowe V, et al. Age-specific and sex-specific prevalence of cerebral β-amyloidosis, tauopathy, and neurodegeneration in cognitively unimpaired individuals aged 50–95 years: a cross-sectional study. Lancet Neurol. 2017;16(6):435–44. 28456479 10.1016/S1474-4422(17)30077-7 PMC5516534

[13] Dale AM, Fischl B, Sereno MI. Cortical surface-based analysis. I. Segmentation and surface reconstruction. Neuroimage. 1999;9(2):179–94. 9931268 10.1006/nimg.1998.0395

[14] Fischl B, Dale AM. Measuring the thickness of the human cerebral cortex from magnetic resonance images. Proc Natl Acad Sci U S A. 2000;97(20):11050–5. 10984517 10.1073/pnas.200033797 PMC27146

[15] La Joie R, Visani AV, Baker SL, Brown JA, Bourakova V, Cha J, et al. Prospective longitudinal atrophy in Alzheimer’s disease correlates with the intensity and topography of baseline tau-PET. Sci Transl Med. 2020;12(524):eaau5732. 31894103 10.1126/scitranslmed.aau5732 PMC7035952

[16] Jack CR, Petersen RC, Xu YC, O’Brien PC, Smith GE, Ivnik RJ, et al. Prediction of AD with MRI-based hippocampal volume in mild cognitive impairment. Neurology. 1999;52(7):1397–1397. 10227624 10.1212/wnl.52.7.1397 PMC2730146

[17] Pini L, Pievani M, Bocchetta M, Altomare D, Bosco P, Cavedo E, et al. Brain atrophy in Alzheimer’s disease and aging. Ageing Res Rev. 2016;30:25–48. 26827786 10.1016/j.arr.2016.01.002

[18] Pinaya WHL, Scarpazza C, Garcia-Dias R, Vieira S, Baecker L, F da Costa P, et al. Using normative modelling to detect disease progression in mild cognitive impairment and Alzheimer’s disease in a cross-sectional multi-cohort study. Sci Rep. 2021;11(1):15746. 34344910 10.1038/s41598-021-95098-0 PMC8333350

[19] Verdi S, Kia SM, Yong KXX, Tosun D, Schott JM, Marquand AF, et al. Revealing Individual Neuroanatomical Heterogeneity in Alzheimer Disease Using Neuroanatomical Normative Modeling. Neurology. 2023;100(24):e2442–53. 37127353 10.1212/WNL.0000000000207298 PMC10264044

[20] Nobis L, Manohar SG, Smith SM, Alfaro-Almagro F, Jenkinson M, Mackay CE, et al. Hippocampal volume across age: Nomograms derived from over 19,700 people in UK Biobank. Neuroimage Clin. 2019;19(23):101904. 10.1016/j.nicl.2019.101904 PMC6603440 31254939

[21] Loreto F, Verdi S, Kia SM, Duvnjak A, Hakeem H, Fitzgerald A, et al. Alzheimer’s disease heterogeneity revealed by neuroanatomical normative modeling. Alzheimers Dement (Amst). 2024;16(1):e12559. 38487076 10.1002/dad2.12559 PMC10937817

[22] Frangou S, Modabbernia A, Williams SCR, Papachristou E, Doucet GE, Agartz I, et al. Cortical thickness across the lifespan: data from 17,075 healthy individuals aged 3–90 years. Hum Brain Mapp. 2022;43(1):431–51. 33595143 10.1002/hbm.25364 PMC8675431

[23] van Rooij D, Anagnostou E, Arango C, Auzias G, Behrmann M, Busatto GF, et al. Cortical and subcortical brain morphometry differences between patients with autism spectrum disorder and healthy individuals across the lifespan: results from the ENIGMA ASD Working Group. Am J Psychiatry. 2018;175(4):359–69. 29145754 10.1176/appi.ajp.2017.17010100 PMC6546164

[24] Barkema P, Rutherford S, Lee HC, Kia SM, Savage H, Beckmann C, et al. Predictive Clinical Neuroscience Portal (PCNportal): instant online access to research-grade normative models for clinical neuroscientists. Wellcome Open Res. 2023;8:326. 37663797 10.12688/wellcomeopenres.19591.2 PMC10474337

[25] Coupé P, Catheline G, Lanuza E, Manjón JV, Alzheimer’s Disease Neuroimaging Initiative. Towards a unified analysis of brain maturation and aging across the entire lifespan: a MRI analysis. Hum Brain Mapp. 2017;38(11):5501–18. 28737295 10.1002/hbm.23743 PMC6866824

[26] Planche V, Manjon JV, Mansencal B, Lanuza E, Tourdias T, Catheline G, et al. Structural progression of Alzheimer’s disease over decades: the MRI staging scheme. Brain Commun. 2022;4(3):fcac109. 35592489 10.1093/braincomms/fcac109 PMC9113086

[27] Planche V, Mansencal B, Manjon JV, Tourdias T, Catheline G, Coupé P. Anatomical MRI staging of frontotemporal dementia variants. Alzheimers Dement. 2023;19(8):3283–94. 36749884 10.1002/alz.12975

[28] Planche V, Mansencal B, Fonov V, Manjon JV, Tourdias T, Bouzigues A, et al. Anatomical progression of genetic frontotemporal lobar degeneration across the lifespan. Brain. 2025;awaf195. 10.1093/brain/awaf195 40424598

[29] Coupé P, Manjón JV, Mansencal B, Tourdias T, Catheline G, Planche V. Hippocampal-amygdalo-ventricular atrophy score: Alzheimer disease detection using normative and pathological lifespan models. Hum Brain Mapp. 2022;43(10):3270–82. 35388950 10.1002/hbm.25850 PMC9188974

[30] Bethlehem RAI, Seidlitz J, White SR, Vogel JW, Anderson KM, Adamson C, et al. Brain charts for the human lifespan. Nature. 2022;604(7906):525–33. 35388223 10.1038/s41586-022-04554-y PMC9021021

[31] Stasinopoulos DM, Rigby RA. Generalized Additive Models for Location Scale and Shape (GAMLSS) in R. J Stat Softw. 2008;23:1–46.

[32] David MCB, Kolanko M, Del Giovane M, Lai H, True J, Beal E, et al. Remote monitoring of physiology in people living with dementia: an observational cohort study. JMIR Aging. 2023;9(6):e43777. 10.2196/43777 PMC10037178 36892931

[33] Beach TG, Monsell SE, Phillips LE, Kukull W. Accuracy of the clinical diagnosis of Alzheimer disease at National Institute on Aging Alzheimer Disease Centers, 2005–2010. J Neuropathol Exp Neurol. 2012;71(4):266–73. 22437338 10.1097/NEN.0b013e31824b211b PMC3331862

[34] Malone IB, Leung KK, Clegg S, Barnes J, Whitwell JL, Ashburner J, et al. Accurate automatic estimation of total intracranial volume: A nuisance variable with less nuisance. Neuroimage. 2015;1(104):366–72. 10.1016/j.neuroimage.2014.09.034 PMC4265726 25255942

[35] Montagnese M, Ebneabbasi A, García-San-Martín N, Pecci-Terroba C, Romero-García R, Morgan SE, et al. Structural Similarity Networks Reveal Brain Vulnerability in Dementia. medRxiv; 2025. p. 2025.06.10.25328978. Available from: https://www.medrxiv.org/content/10.1101/2025.06.10.25328978v1. Cited 2025 Aug 23. 10.1002/alz.70973 PMC12741943 41451870

[36] Visualization of Brain Statistics With R Packages ggseg and ggseg3d - Athanasia M. Mowinckel, Didac Vidal-Piñeiro, 2020. Available from: https://journals.sagepub.com/doi/full/10.1177/2515245920928009. Cited 2024 Feb 19.

[37] Ashton NJ, Brum WS, Di Molfetta G, Benedet AL, Arslan B, Jonaitis E, et al. Diagnostic accuracy of a plasma phosphorylated Tau 217 immunoassay for alzheimer disease pathology. JAMA Neurol. 2024;81(3):255–63. 38252443 10.1001/jamaneurol.2023.5319 PMC10804282

[38] Lee J, Ward T, Harrison T, Landau S. ADNI Tau PET Processing Methods. 2023. Available from: https://ida.loni.usc.edu/login.jsp?project=ADNI

[39] Marshall CR, Hardy CJD, Volkmer A, Russell LL, Bond RL, Fletcher PD, et al. Primary progressive aphasia: a clinical approach. J Neurol. 2018;265(6):1474–90. 29392464 10.1007/s00415-018-8762-6 PMC5990560

[40] Rohrer JD, Rosen HJ. Neuroimaging in frontotemporal dementia. Int Rev Psychiatry. 2013;25(2):221–9. 23611351 10.3109/09540261.2013.778822

[41] Schöll M, Lockhart SN, Schonhaut DR, O’Neil JP, Janabi M, Ossenkoppele R, et al. PET Imaging of Tau Deposition in the Aging Human Brain. Neuron. 2016;89(5):971–82. 26938442 10.1016/j.neuron.2016.01.028 PMC4779187

[42] Schöll M, Ossenkoppele R, Strandberg O, Palmqvist S, Swedish BioFINDER study, Jögi J, et al. Distinct 18F-AV-1451 tau PET retention patterns in early- and late-onset Alzheimer’s disease. Brain. 2017;140(9):2286–94. 29050382 10.1093/brain/awx171

[43] Struyfs H, Sima DM, Wittens M, Ribbens A, Pedrosa de Barros N, Phan TV, et al. Automated MRI volumetry as a diagnostic tool for Alzheimer’s disease: Validation of icobrain dm. Neuroimage Clin. 2020;26:102243. 10.1016/j.nicl.2020.102243 PMC7082216 32193172

[44] Ross DE, Ochs AL, Seabaugh JM, Shrader CR. Man versus machine: comparison of radiologists’ interpretations and NeuroQuant® volumetric analyses of brain MRIs in patients with traumatic brain injury. J Neuropsychiatry Clin Neurosci. 2013;25(1):32–9. 23487191 10.1176/appi.neuropsych.11120377 PMC7185228

[45] Bedford SA, Seidlitz J, Bethlehem RAI. Translational potential of human brain charts. Clin Transl Med. 2022;12(7):e960. 35858047 10.1002/ctm2.960 PMC9299572

[46] van Dyck CH, Swanson CJ, Aisen P, Bateman RJ, Chen C, Gee M, et al. Lecanemab in early Alzheimer’s disease. N Engl J Med. 2023;388(1):9–21. 36449413 10.1056/NEJMoa2212948

[47] Sims JR, Zimmer JA, Evans CD, Lu M, Ardayfio P, Sparks J, et al. Donanemab in Early Symptomatic Alzheimer Disease: The TRAILBLAZER-ALZ 2 Randomized Clinical Trial. JAMA. 2023;330(6):512–27. 37459141 10.1001/jama.2023.13239 PMC10352931

[48] Filip P, Bednarik P, Eberly LE, Moheet A, Svatkova A, Grohn H, et al. Different FreeSurfer versions might generate different statistical outcomes in case-control comparison studies. Neuroradiology. 2022;64(4):765–73. 34988592 10.1007/s00234-021-02862-0 PMC8916973

[49] Billot B, Greve DN, Puonti O, Thielscher A, Van Leemput K, Fischl B, et al. SynthSeg: Segmentation of brain MRI scans of any contrast and resolution without retraining. Med Image Anal. 2023;1(86):102789. 10.1016/j.media.2023.102789 PMC10154424 36857946

[50] Bothongo PLK, Jitlal M, Parry E, Waters S, Foote IF, Watson CJ, et al. Dementia risk in a diverse population: a single-region nested case-control study in the East End of London. The Lancet Regional Health - Europe. 2022;1(15):100321. 10.1016/j.lanepe.2022.100321 PMC9088197 35558994

[51] Henrich J, Heine SJ, Norenzayan A. The weirdest people in the world? Behav Brain Sci. 2010;33(2–3):61–83 (discussion 83-135). 20550733 10.1017/S0140525X0999152X

[52] Alosco ML, Mian AZ, Buch K, Farris CW, Uretsky M, Tripodis Y, et al. Structural MRI profiles and tau correlates of atrophy in autopsy-confirmed CTE. Alzheimers Res Ther. 2021;13(1):193. 34876229 10.1186/s13195-021-00928-y PMC8653514

[53] Mak E, Su L, Williams GB, Firbank MJ, Lawson RA, Yarnall AJ, et al. Baseline and longitudinal grey matter changes in newly diagnosed Parkinson’s disease: ICICLE-PD study. Brain. 2015;138(Pt 10):2974–86. 26173861 10.1093/brain/awv211 PMC4671477

[54] Machts J, Cardenas-Blanco A, Acosta-Cabronero J, Kaufmann J, Loewe K, Kasper E, et al. Prefrontal cortical thickness in motor neuron disease. Neuroimage Clin. 2018;2(18):648–55. 10.1016/j.nicl.2018.03.002 PMC5987868 29876256

[55] Li K, Luo X, Zeng Q, Liu X, Li J, Zhong S, et al. Gray matter structural covariance networks patterns associated with autopsy-confirmed LATE-NC compared to Alzheimer’s disease pathology. Neurobiol Dis. 2023;1(189):106354. 10.1016/j.nbd.2023.106354 37977431

[56] Dorfschmidt L, White S, Gardner M, Bedford S, Ball G, Edwards AD, et al. Charting structural brain asymmetry across the human lifespan. bioRxiv; 2025. p. 2025.07.21.665924. Available from: https://www.biorxiv.org/content/10.1101/2025.07.21.665924v1. Cited 2025 Aug 12.

[57] Di Biase MA, Tian YE, Bethlehem RAI, Seidlitz J, Alexander-Bloch Aaron F, Yeo BTT, et al. Mapping human brain charts cross-sectionally and longitudinally. Proc Natl Acad Sci U S A. 2023;120(20):e2216798120. 10.1073/pnas.2216798120 PMC10193972 37155868

