# Supplementary material for: Generalizable MRI normative modelling to detect age-inappropriate neurodegeneration
Source: Alzheimers Res Ther. 2025 Nov 12;17:244. doi: 10.1186/s13195-025-01872-x (PMC12613731; doi:10.1186/s13195-025-01872-x)
Supplement: Supplementary file 1 — Supplementary Material 1. [file 13195_2025_1872_MOESM1_ESM.docx]

# **Supplementary material**

| **ROI** | **AUC** | **AUC sensitivity analysis** |
| --- | --- | --- |
|  |  |  |
| Amygdala -  left | 0.82 | 0.82 |
| Amygdala - right | 0.79 | 0.79 |
| Hippocampus - left | 0.79 | 0.80 |
| Hippocampus - right | 0.78 | 0.77 |
| Entorhinal -  left | 0.70 | 0.71 |
| Entorhinal - right | 0.72 | 0.73 |
| Inferior temporal - left | 0.68 | 0.71 |
| Inferior temporal - right | 0.68 | 0.70 |
| Middle temporal - left | 0.71 | 0.73 |
| Middle temporal - right | 0.71 | 0.74 |
| Fusiform -  left | 0.70 | 0.72 |
| Fusiform -  right | 0.71 | 0.74 |
| Precuneus -  left | 0.69 | 0.73 |
| Precuneus - right | 0.70 | 0.73 |
| Inferior parietal - left | 0.72 | 0.75 |
| Inferior parietal - right | 0.71 | 0.74 |
| Combined | 0.86 | 0.87 |

*Supplementary Table 1. Sensitivity analysis of regional BrainChart centile score classification performance in the NACC dataset after excluding scans with Euler index values greater than 2 median absolute deviations above the study median.*

| Rank | Hemisphere | FreeSurfer region | AUC |  | Rank | Hemisphere | FreeSurfer region | AUC |
| --- | --- | --- | --- | --- | --- | --- | --- | --- |
| 1 | Right | Entorhinal* | 0.72 |  | 35 | Right | Lateral occipital | 0.66 |
| 2 | Left | Inferior parietal* | 0.71 |  | 36 | Right | Posterior cingulate | 0.66 |
| 3 | Left | Middle temporal* | 0.71 |  | 37 | Right | Precentral | 0.65 |
| 4 | Right | Inferior parietal* | 0.71 |  | 38 | Left | Lateral occipital | 0.65 |
| 5 | Right | Fusiform* | 0.71 |  | 39 | Right | Paracentral | 0.65 |
| 6 | Right | Middle temporal* | 0.71 |  | 40 | Left | Rostral anterior cingulate | 0.65 |
| 7 | Left | Superior temporal | 0.70 |  | 41 | Left | Rostral middle frontal | 0.65 |
| 8 | Left | Supramarginal | 0.70 |  | 42 | Right | Lateral orbitofrontal | 0.65 |
| 9 | Left | Fusiform* | 0.70 |  | 43 | Right | Postcentral | 0.65 |
| 10 | Right | Precuneus* | 0.70 |  | 44 | Left | Caudal middle frontal | 0.65 |
| 11 | Right | Isthmus cingulate | 0.70 |  | 45 | Left | Posterior cingulate | 0.64 |
| 12 | Right | Superior temporal | 0.70 |  | 46 | Right | Rostral anterior cingulate | 0.64 |
| 13 | Right | Supramarginal | 0.70 |  | 47 | Right | Medial orbitofrontal | 0.64 |
| 14 | Left | Entorhinal* | 0.70 |  | 48 | Left | Precentral | 0.64 |
| 15 | Right | Insula | 0.69 |  | 49 | Left | Postcentral | 0.64 |
| 16 | Left | Isthmus cingulate | 0.69 |  | 50 | Left | Medial orbitofrontal | 0.63 |
| 17 | Left | Precuneus* | 0.69 |  | 51 | Left | Transverse temporal | 0.63 |
| 18 | Right | Temporal pole | 0.69 |  | 52 | Left | Pars triangularis | 0.63 |
| 19 | Right | Pars opercularis | 0.68 |  | 53 | Right | Cuneus | 0.63 |
| 20 | Left | Insula | 0.68 |  | 54 | Right | Pars orbitalis | 0.63 |
| 21 | Right | Caudal middle frontal | 0.68 |  | 55 | Left | Lingual | 0.63 |
| 22 | Right | Inferior temporal* | 0.68 |  | 56 | Left | Lateral orbitofrontal | 0.63 |
| 23 | Right | Pars triangularis | 0.68 |  | 57 | Right | Transverse temporal | 0.63 |
| 24 | Right | Rostral middle frontal | 0.68 |  | 58 | Right | Lingual | 0.62 |
| 25 | Right | Superior frontal | 0.68 |  | 59 | Left | Frontal pole | 0.62 |
| 26 | Left | Inferior temporal* | 0.68 |  | 60 | Left | Pars opercularis | 0.62 |
| 27 | Left | Banks of the superior temporal sulcus | 0.68 |  | 61 | Left | Pars orbitalis | 0.62 |
| 28 | Right | Banks of the superior temporal sulcus | 0.67 |  | 62 | Left | Paracentral | 0.61 |
| 29 | Left | Superior parietal | 0.67 |  | 63 | Left | Cuneus | 0.61 |
| 30 | Left | Temporal pole | 0.67 |  | 64 | Right | Frontal pole | 0.61 |
| 31 | Left | Parahippocampal | 0.67 |  | 65 | Right | Pericalcarine | 0.58 |
| 32 | Right | Parahippocampal | 0.67 |  | 66 | Left | Pericalcarine | 0.58 |
| 33 | Left | Superior frontal | 0.67 |  | 67 | Right | Caudal anterior cingulate | 0.57 |
| 34 | Right | Superior parietal | 0.67 |  | 68 | Left | Caudal anterior cingulate | 0.56 |

*Supplementary table 2. BrainChart regional centile scores applied to 351 patients with pathologically confirmed Alzheimer’s disease from the National Alzheimer’s Coordinating Center (NACC) dataset. AUC rank for all 68 cortical regions from the FreeSurfer Desikan-Killiany cortical atlas. * a priori Alzheimer’s disease cortical thickness signature regions*

| **ROI** | **AUC** | **Youden-Index cut off** | **Youden-Index**  **cut off** | | **<0 25** | | **<0.10** | | **<0.05** | | **<0.01** | |
| --- | --- | --- | --- | --- | --- | --- | --- | --- | --- | --- | --- | --- |
|  |  |  | Sens | Spec | Sens | Spec | Sens | Spec | Sens | Spec | Sens | Spec |
| Amygdala -  left | 0.82 | 0.26 | 70% | 83% | 68% | 84% | 51% | 93% | 39% | 96% | 20% | 98% |
| Amygdala - right | 0.79 | 0.26 | 64% | 83% | 64% | 83% | 40% | 95% | 29% | 96% | 13% | 98% |
| Hippocampus - left | 0.79 | 0.26 | 66% | 82% | 66% | 83% | 43% | 95% | 35% | 96% | 19% | 97% |
| Hippocampus - right | 0.78 | 0.23 | 64% | 79% | 65% | 78% | 43% | 93% | 32% | 96% | 19% | 97% |
| Entorhinal -  left | 0.70 | 0.33 | 64% | 68% | 56% | 74% | 38% | 84% | 27% | 89% | 13% | 95% |
| Entorhinal - right | 0.72 | 0.37 | 68% | 65% | 57% | 73% | 37% | 87% | 29% | 91% | 11% | 97% |
| Inferior temporal - left | 0.68 | 0.39 | 63% | 68% | 49% | 77% | 28% | 87% | 21% | 91% | 10% | 94% |
| Inferior temporal - right | 0.68 | 0.41 | 66% | 65% | 47% | 77% | 30% | 87% | 23% | 91% | 12% | 95% |
| Middle temporal - left | 0.71 | 0.34 | 65% | 69% | 56% | 77% | 37% | 87% | 25% | 91% | 8% | 93% |
| Middle temporal - right | 0.71 | 0.42 | 69% | 66% | 55% | 78% | 36% | 87% | 25% | 90% | 9% | 93% |
| Fusiform -  left | 0.70 | 0.30 | 60% | 72% | 56% | 77% | 36% | 87% | 25% | 90% | 13% | 94% |
| Fusiform -  right | 0.71 | 0.47 | 70% | 63% | 51% | 79% | 35% | 87% | 25% | 90% | 16% | 94% |
| Precuneus -  left | 0.69 | 0.30 | 61% | 70% | 52% | 74% | 31% | 88% | 20% | 90% | 8% | 96% |
| Precuneus - right | 0.70 | 0.45 | 72% | 58% | 55% | 74% | 34% | 88% | 19% | 92% | 7% | 96% |
| Inferior parietal - left | 0.72 | 0.38 | 70% | 66% | 55% | 75% | 40% | 86% | 26% | 90% | 8% | 95% |
| Inferior parietal - right | 0.71 | 0.42 | 73% | 62% | 55% | 76% | 33% | 86% | 23% | 91% | 9% | 95% |

*Supplementary table 3. BrainChart regional centile scores applied to 351 patients with pathologically confirmed Alzheimer’s disease from the National Alzheimer’s Coordinating Center (NACC) dataset. AUC, Optimal cut point values determined using a Youden-Index approach with kernel smoothed densities and sensitivity and specificity over a range of centile scores for the left and right hemisphere of seven regions previously proposed as biomarkers of neurodegeneration in Alzheimer’s disease for differentiating pathologically confirmed AD from propensity-matched cognitively normal participants are shown. Key: ROI = region of interest, Sens = true positive rate (sensitivity), Spec = true negative rate (specificity).*

| bvFTD compared to propensity-matched controls | | | | bvFTD compared to propensity-matched pathological AD | | | |
| --- | --- | --- | --- | --- | --- | --- | --- |
| Rank | Hemisphere | FreeSurfer region | AUC | Rank | Hemisphere | FreeSurfer region | AUC |
| 1 | Left | Superior frontal | 0.91 | 1 | Left | Superior frontal | 0.79 |
| 2 | Left | Caudal middle frontal | 0.88 | 2 | Left | Caudal middle frontal | 0.78 |
| 3 | Right | Hippocampus | 0.87 | 3 | Left | Pars opercularis | 0.76 |
| 4 | Left | Hippocampus | 0.87 | 4 | Right | Superior Frontal | 0.76 |
| 5 | Left | Middle temporal | 0.86 | 5 | Left | Lateral orbitofrontal | 0.75 |
| 6 | Right | Middle temporal | 0.86 | 6 | Left | Rostral middle frontal | 0.75 |
| 7 | Right | Superior Frontal | 0.86 | 7 | Left | Pars orbitalis | 0.74 |
| 8 | Left | Pars opercularis | 0.85 | 8 | Left | Pars triangularis | 0.74 |
| 9 | Left | Rostral middle frontal | 0.85 | 9 | Left | Medial orbitofrontal | 0.73 |
| 10 | Right | Parahippocampal | 0.84 | 10 | Right | Medial orbitofrontal | 0.72 |

*Supplementary Table 4. BrainChart regional centile scores applied to 49 behavioural frontotemporal dementia patients from NIFD. Discrimination from propensity mathed pathologically confirmed Alzheimer’s disease. AUC rank for top 10 regions.*

| PPA-SV v controls | | | | PPA-PNFA v controls | | | |
| --- | --- | --- | --- | --- | --- | --- | --- |
| Rank | Hemisphere | FreeSurfer region | AUC | Rank | Hemisphere | FreeSurfer region | AUC |
| 1 | Left | Amygdala | 1.00 | 1 | Left | Pars triangularis | 0.80 |
| 2 | Left | Entorhinal | 1.00 | 2 | Left | Precentral | 0.79 |
| 3 | Left | Hippocampus | 1.00 | 3 | Left | Caudal middle frontal | 0.79 |
| 4 | Left | Temporal pole | 1.00 | 4 | Left | Superior frontal | 0.78 |
| 5 | Left | Inferior temporal | 1.00 | 5 | Right | Superior frontal | 0.76 |
| 6 | Left | Superior temporal | 1.00 | 6 | Left | Superior frontal | 0.75 |
| 7 | Left | Middle temporal | 0.99 | 7 | Left | Parahippocampal | 0.76 |
| 8 | Left | Entorhinal | 0.98 | 8 | Left | Pars opercularis | 0.75 |
| 9 | Right | Temporal pole | 0.97 | 9 | Right | Pars opercularis | 0.74 |
| 10 | Left | Fusiform | 0.97 | 10 | Right | Transverse temporal | 0.73 |

*Supplementary Table 5. BrainChart regional centile scores applied to PPA patients from NIFD. Discrimination from normal controls. AUC rank for top 10 regions.*

| PPA-SV v PPA-PNFA | | | | PPA-PNFA v PPA-SV | | | |
| --- | --- | --- | --- | --- | --- | --- | --- |
| Rank | Hemisphere | FreeSurfer region | AUC | Rank | Hemisphere | FreeSurfer region | AUC |
| 1 | Right | Temporal pole | 0.98 | 1 | Left | Precentral | 0.69 |
| 2 | Left | Amygdala | 0.97 | 2 | Left | Caudal middle frontal | 0.68 |
| 3 | Left | Temporal pole | 0.96 | 3 | Left | Pars triangularis | 0.67 |
| 4 | Left | Entorhinal | 0.95 | 4 | Right | Superior frontal | 0.66 |
| 5 | Left | Hippocampus | 0.95 | 5 | Left | Superior frontal | 0.65 |
| 6 | Right | Entorhinal | 0.95 | 6 | Right | Posterior cingulate | 0.64 |
| 7 | Left | Fusiform | 0.95 | 7 | Right | Caudal middle frontal | 0.64 |
| 8 | Left | Superior temporal | 0.94 | 8 | Right | Precentral | 0.63 |
| 9 | Left | Middle temporal | 0.93 | 9 | Right | Transverse Temporal | 0.62 |
| 10 | Left | Inferior temporal | 0.93 | 10 | Right | Pars opercularlis | 0.62 |

*Supplementary Table 6. BrainChart regional centile scores applied to PPA patients from NIFD. Discrimination between PPA-SV and PPA-PNFA phenotypes. AUC rank for top 10 regions.*
